# Oxygen - a limiting factor for brain recovery

**DOI:** 10.1186/s13054-015-1034-2

**Published:** 2015-09-01

**Authors:** Amir Hadanny, Shai Efrati

**Affiliations:** Sagol Center for Hyperbaric Medicine and Research, Assaf Harofeh Medical Center, Zerifin, 70300 Israel; Sackler School of Medicine, Tel-Aviv University, Klachkin 25, Tel Aviv, 69978 Israel; Research and Development Unit, Assaf Harofeh Medical Center, Zerifin, 70300 Israel; Sagol School of Neuroscience, Tel-Aviv University, Klachkin 25, Tel Aviv, 69978 Israel

## Abstract

Effective brain metabolism is highly dependent on a narrow therapeutic window of oxygen. In major insults to the brain (e.g., intracerebral hemorrhage), a slight decrease in oxygen supply, as occurs in a hypobaric environment at high altitude, has devastating effects on the injured brain tissue. Conversely, increasing brain oxygenation, by the use of hyperbaric oxygen therapy, can improve brain metabolism and its dependent regenerative processes.

## Introduction

Intracerebral hemorrhage (ICH) carries significant mortality and morbidity. At high altitude, the prognosis of ICH is worsened and one of the possible explanations relates to the lower oxygen concentration. In a recent article by Zhu et al. [1], this claim was challenged by exposing minipigs with ICH to hypo-, normo-, and hyperbaric environments. In a hypobaric setting, compared with a normobaric environment, brain tissue oxygenation (PbTO_2_), brain metabolism (lactate, lactate–pyruvate ratio, and glutamate), and neurological scores were significantly worsened. In contrast, a significant improvement in all metabolic parameters was achieved when hyperbaric oxygen was applied, resulting in significant improvement in neurological functions.

## Commentary

From a thermodynamic perspective, the brain is a unique organ in our body. The brain comprises only about 2 % of the body’s total weight but uses about 15 % of total cardiac output, 20 % of total oxygen supply, 25–30 % of total body glucose consumption, and 30 % of total body energy consumption. There is a linear relationship between normal arterial oxygen partial pressure (pO_2_) and PbTO_2_ [2]. Under normal normobaric conditions, arterial partial pressure of oxygen (paO_2_) is approximately 90 mm Hg, whereas cerebrovenous pO_2_ pressure is about 35 mm Hg. Because oxygen is continuously consumed by the brain, PbTO_2_ is a continuum with values ranging from 90 mm Hg, very close to capillaries, to much less than 34 mm Hg in more distal regions [2]. PbTO_2_ values below 15 mm Hg indicate ischemia, and values below 5 mm Hg indicate brain necrosis.

The minimal paO_2_ concentrations required for supplying sufficient amounts of intracellular oxygen for effective neurological functioning are unknown. However, we do know that reduction of paO_2_ to 65 mm Hg results in impaired ability of the brain to perform complex tasks. At 55 mm Hg, short-term memory is impaired, and paO_2_ of 30 mm Hg causes loss of consciousness [2]. Further decrease in paO_2_ levels may result in tissue infarction, depending on the duration of hypoxia.

Under normal healthy conditions, brain metabolism reaches the upper limit of oxygen consumption, which makes it dependent on cerebral blood flow (CBF). At each time point, the CBF shifts to more active regions (task-dependent) at the expense of other, less active regions. These physiological changes in CBF can be easily seen in functional magnetic resonance imaging. Major insults to the brain, such as ICH, ischemic stroke, or traumatic brain injury, compromise CBF and further decrease the oxygen delivery to the “non-active”, injured brain tissue. In a hypobaric environment, oxygen delivery decreases below the minimal metabolic needs. Zhu et al. [1] have shown that in ICH in a hypobaric environment, when paO_2_ reached 63–66 mm Hg and the diffusion distance for oxygen delivery from microvasculature to brain tissue was expanded because of edema, extravascular blood accumulation, and structural damage of adjacent tissue, the PbTO_2_ is as low as 9–14 mm Hg. Consequently, achieving higher tissue oxygen tension and paO_2_ is crucial for maintaining sufficient oxygenation of the damaged brain tissue.

Clinical studies published this year present convincing evidence that hyperbaric oxygen therapy (HBOT) can be the coveted neurotherapeutic method for brain repair [3–6]. HBOT is a treatment in which oxygen-enriched air (up to 100 %) is administrated to patients in a chamber where the pressure is elevated above 1 ATA (one atmosphere absolute, which is the ambient atmospheric pressure at sea level). Increasing the plasma oxygen concentration with hyperbaric oxygenation is a potent means of delivering sufficient oxygen to the brain for tissue repair. HBOT might initiate a cellular and vascular repair mechanism and improve cerebral vascular flow [3]. At the cellular level, HBOT can improve mitochondrial function (in both neurons and glial cells) and cellular metabolism, improve blood–brain barrier and inflammatory reactions, reduce apoptosis, alleviate oxidative stress, increase levels of neurotrophins and nitric oxide, and upregulate axon guidance agents. Moreover, the effects of HBOT on neurons can be mediated indirectly by glial cells, including astrocytes. HBOT may also promote neurogenesis of the endogenous neural stem cells. At the vascular level, HBOT was found to have a role in initiation or facilitation of angiogenesis (or both) and cell proliferation needed for axonal regeneration. It is possible that HBOT enables all of the above-mentioned metabolic changes simply by supplying the missing energy/oxygen needed for those regeneration processes.

## Conclusions

In summary, oxygen is crucial for effective metabolism in the brain. As was well proven by Zhu et al., the therapeutic window is very narrow and any slight decrease in oxygen delivery has devastating effects on the injured brain tissue. HBOT may well be the sought-after tool, both effective and simple, for improving PbTO_2_ and brain metabolism needed for the regenerative processes.

## Abbreviations

CBF: Cerebral blood flow
HBOT: Hyperbaric oxygen therapy
ICH: Intracerebral hemorrhage
paO_2_: Arterial partial pressure of oxygen
PbTO_2_: Brain tissue oxygenation
pO_2_: Oxygen partial pressure
